# Pictogram Is a Valid Instrument to Classify At-Risk Adult Population Based on Abdominal Obesity: Results from Pars Cohort Study

**DOI:** 10.34172/aim.2022.60

**Published:** 2022-06-01

**Authors:** Alireza Kamalipour, Amirhossein Roshanshad, Mahdi Nalini, Jafar Hassanzadeh, Reza Malekzadeh, Fatemeh Malekzadeh, Hossein Poustchi, Abdullah Gandomkar, Alireza Salehi, Hossein Molavi Vardanjani

**Affiliations:** ^1^MPH Department, School of Medicine, Shiraz University of Medical Sciences, Shiraz, Iran; ^2^Hamilton Glaucoma Center, Shiley Eye Institute, Viterbi Family Department of Ophthalmology, University of California, San Diego, California, United States; ^3^Student Research Committee, Shiraz University of Medical Sciences, Shiraz, Iran; ^4^Cardiovascular Research Center, Kermanshah University of Medical Sciences, Kermanshah, Iran; ^5^Department of Epidemiology, Shiraz University of Medical Sciences, Shiraz, Iran; ^6^Liver, Pancreatic, and Biliary Diseases Research Center, Digestive Diseases Research Institute, Tehran University of Medical Sciences, Tehran, Iran; ^7^Digestive Disease Research Center, Digestive Research Institute, Shariati Hospital, Tehran University of Medical Science, Tehran, Iran; ^8^Non-Communicable Disease Research Center, Shiraz University of Medical Sciences, Shiraz, Iran; ^9^MPH Department, School of Medicine, Research Center for Traditional Medicine and History of Medicine, Shiraz University of Medical Sciences, Shiraz, Iran

**Keywords:** Abdominal obesity, Pictogram, Waist circumference, Waist-height ratio, Waist-hip ratio

## Abstract

**Background:**

Abdominal obesity is associated with increased risk of myocardial infarction and death events. Thus, obtaining data on the status of abdominal obesity is important in risk factor assessment and prevention of non-communicable diseases. This study aimed to evaluate the validity of using pictograms to classify abdominal obesity indices (waist circumference [WC], waist-hip ratio [WHR], and waist-height ratio [WHtR]) into normal and at-risk categories and determine the effects of demographic characteristics on this validity.

**Methods:**

This cross-sectional study used data from Pars Cohort Study (PCS). Participants chose the most similar pictogram scores to their body size at 15, 30 years, and current age. Optimal normal/at-risk cut-off values for pictograms were calculated using sensitivity/specificity plots. Receiver operating characteristic curves were used to evaluate the validity of pictograms. Validity measures were analyzed across different subgroups of demographic characteristics.

**Results:**

A total of 9263 participants (46% males) were included in the study. The estimated area under the curves were 84% for WC, 77% for WHR, and 89% for WHtR in males, and 84% for WC, 73% for WHR, and 90% for WHtR in females. Optimal pictogram cutoffs to classify central obesity for WC, WHR, and WHtR were 4, 4, and 5 in males and 4, 4, and 6 in females, respectively. The majority of demographic characteristics were not associated with the validity of pictograms.

**Conclusion:**

Using pictograms to determine normal and at-risk categories of abdominal obesity indices is valid among adult population with a wide range of demographic characteristics. However, the results need to be interpreted with caution in those with a positive history of weight fluctuation.

## Introduction

 Maldistribution of total body fat is associated with adverse metabolic and cardiac outcomes.^1,2^ Visceral body fat increases the risk of insulin resistance and metabolic syndrome.^3^ Furthermore, previous studies show that correlations of abdominal obesity indices (waist circumference [WC], waist-hip ratio [WHR], and waist-height ratio [WHtR]) with myocardial infarction and death events are higher than the correlation of body mass index (BMI) with these events.^4,5^ Thus, obtaining data on the status of abdominal obesity is important in risk factor assessment and prevention of non-communicable diseases.^6^

 Evaluation of abdominal obesity indices in field studies can be problematic, especially in low-resource settings, due to several reasons. First, it is expensive and time-consuming to provide the required instruments for accurate measurement on a large scale.^7^ Second, measuring these indices requires experienced technicians. Finally, in line with the development of telemedicine, alternative assessment methods should be provided that do not require the physical presence of the participants.

 Body shape pictogram (silhouette) can be a potential alternative of using direct measurements to classify abdominal obesity status. Pictograms are sets of body images ranging from very lean to extremely obese for each gender.^8,9^ Participants are required to select the picture of the pictogram most similar to their body shape. This instrument has been previously used in several studies for the main purpose of BMI classification.^10,11^ A previously designed pictogram by Stunkard et al^11^ has also been validated to estimate BMI in a study conducted in northern Iran.^9^

 To the best of our knowledge, the validity of using pictograms in the classification of abdominal obesity status has not been previously studied in the Iranian population. In this study, we aimed to evaluate the validity of using pictograms to classify WC, WHR, and WHtR categories and also to determine the demographic factors that may alter its classification accuracy in a representative adult population from southern Iran.

## Materials and Methods

###  Study Design and Population

 This cross-sectional study was conducted using the baseline data obtained from Pars Cohort Study (PCS). PCS is an ongoing population-based prospective study that started in the fall of 2012 in southern Iran, on a semi-urban multi-ethnic population aged between 40 and 75 years. Details of the study design are published elsewhere.^12^

###  Variable Measurement

 PCS data were collected using personal interviews, physical examination, and biological sampling based on the study manuals using standardized and calibrated tools by well-experienced personnel. In this study, we used data on age, gender, marital status, ethnicity, education, physical activity, list of household appliances, assets and entertainments, and anthropometric measures including a single measure for height, weight, waist, and hip circumferences. Anthropometric measurements were done when participants wore light clothing, emptied their pockets completely, and did not wear shoes. These measurements were performed once. Height, WC, and hip circumference were measured to the nearest centimeter. WC was measured at the midpoint between the lower margin of the last palpable rib and the top of the iliac crest. The widest portion of the buttocks was considered for measurement of the hip circumference.^13^ Participants’ weight was measured to the nearest 100 g. One kilogram of measured weight was deducted, equivalent to the average weight of light clothing.

**Figure 1:**
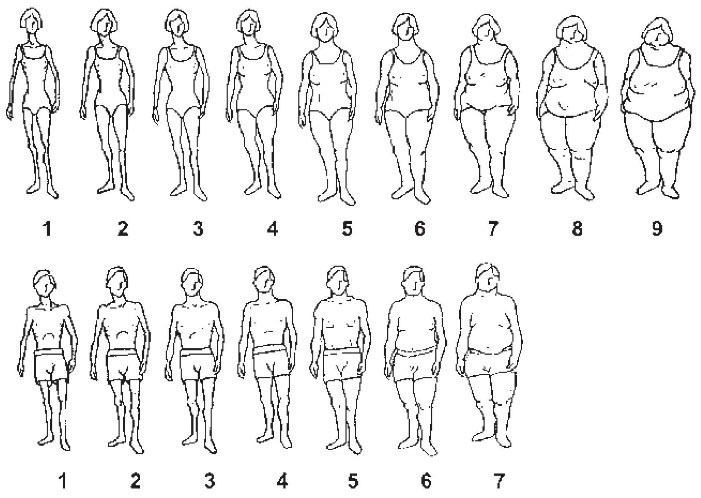
Body Image Pictogram Used in the Pars Cohort Study.

 Data on the individual perception of body size and appearance were previously collected using a self-reported questionnaire. The body silhouettes used in this study were previously designed by Stunkard et al.^11^ The participants were asked to choose their body image perception at the ages of 15 and 30 years, and at the time of the interview. The pictograms showed increasing body size in ascending order and included 7 and 9 pictures for male and female individuals, respectively (Figure 1).

###  Data Cleaning and Preparation

 The data were assessed for internal and external consistency and also rechecked with the hard copy if needed. Cleaned data were prepared for the analyses.

 Socioeconomic status (SES) was determined by assets analysis using multiple correspondence analysis of data on the properties. Several items were used to determine the SES of the participants, including phone at home, cell phone, washing machine, dishwasher, microwave device, camcorder, car, household residential area, number of rooms per household members, the main cooling devices and the main cooking device. Then, the participants were categorized into four ordered SES groups.^14^ Physical activity (PA) was assessed based on the Metabolic Equivalent of Task (MET) score. We implemented International Physical Activity Questionnaire (IPAQ) and used the standard method for its analysis. Then, MET scores were divided into three thirties, named low, medium and high PA.^15^ The long-term weight cycling variable was defined based on the selected pictograms at three cut-points of 15 years, 30 years, and current age. Individuals who selected the 30-year pictogram as their lowest or highest score (demonstrating a fluctuation in the pattern of pictograms over time) were labeled as cyclers. The remaining participants were classified as non-cyclers.

 BMI was divided into underweight/normal (< 25), overweight (≥ 25 and < 30), and obese (≥ 30) similar to the cut-off used by Keshtkar et al.^9^ Age was categorized into three separate groups of < 50 years, ≥ 50 and < 60 years, and ≥ 60 years. Education and ethnicity variables were classified into 3 groups each (illiterate, ≤ 12 years of education, and university degree), and (Persian, Turk, and others), respectively. WC was categorized into two groups of at-risk and normal based on a limit of ≥ 90 cm for both genders.^16^ WHR was classified into two groups of at-risk and normal with a cut-off value of ≥ 0.95 for males and ≥ 0.90 for females.^17^ Finally, WHtR ≥ 0.51 for both genders was classified as at-risk based on a previous study in the Iranian population.^18,19^

###  Statistical Analysis

 Descriptive statistics including frequencies, median and interquartile range (IQR) were estimated. Gender-specific directly age-standardized proportions of at-risk WC, WHR and, WHtR along with their 95% confidence intervals (CIs) were estimated using world standard population 2000–2025. The linearity assumption for the association between successive levels of the pictogram’s ordinal scores and different central obesity indices was evaluated for each gender. Pearson’s correlation between the pictogram score and different measures of abdominal obesity was assessed for each gender.

 Previously defined cut-off values for WC, WHR, and WHtR in the Iranian population were used as gold standard measures for classifying people into normal and at-risk categories of central obesity. Sensitivity and specificity values and their 95% CI were calculated for each of the pictogram pictures to discriminate participants with at-risk values of WC, WHR, and WHtR from normal values. The best cut-off value was determined based on sensitivity/specificity plots and optimized Youden Index which is defined as sensitivity + specificity -1.^20^ The classification accuracy of using pictogram to divide people into normal and at-risk categories based on each central obesity measure was assessed using the area under the receiver operating characteristic curve (AUC) [%, 95% CI] obtained from logistic regression models. To assess the effect of background variables of the target population on the validity of pictograms, stratified statistical analyses were done using the test for the equality of AUCs based on an algorithm suggested by DeLong et al.^21^ Stata software version 14.1 (College Station, TX: Stata Corp LLC) was used and a *P* value < 0.05 was considered as statistically significant.

## Results

 A total of 9263 participants including 4276 males (46%) were included in the study. Forty-five percent were younger than 50 years. Age-standardized proportions of at-risk WC, WHR, and WHtR were 54% (95% CI: 53, 55), 63% (95% CI: 62, 64), and 9% (95% CI: 9, 10), respectively. Details of the sociodemographic characteristics of the study population are described in Table 1.

**Table 1:** Demographics of the Overall and At-Risk Population Based on WC, WHR, and WHtR

**Variables**	**Overall** **(n=9264)** **No. (%)**	**At Risk**		
		**WC** **No (%; 95%CI)**	**WHR** **No (%; 95%CI)**	**WHtR** **No (%; 95%CI)**
Age				
Mean age (SD)	52.63(9.68)	52.48(9.36)	53.65(9.65)	50.89(8.26)
< 50	4216(46)	2294(55; 53, 56)	2261(54; 52,55)	477(11; 10, 12)
50–59	2808(30)	1600(57; 55, 59)	1830(66; 64, 67)	288(10; 9, 11)
> 59	2240(24)	1195(54; 51, 56)	1564(70; 68, 72)	161(7; 6, 8)
Gender				
Female	4987(54)	3092(62; 61, 64)	3741(75; 74, 77)	552(11; 10, 12)
Male	4276(46)	1997(47; 45, 48)	1914(45; 44, 46)	374(9; 8, 10)
Ethnicity				
Persian	5216(56)	2996(58; 56, 59)	3281(63; 62, 64)	607(12; 11, 13)
Turk	3596(39)	1809(51; 49, 52)	2060(58; 56, 59)	258(7; 6, 8)
Other	451(5)	284(63; 59, 67)	314(70; 65, 74)	61(14; 11, 17)
BMI				
Underweight/normal	4091(44)	597(15; 14, 16)	1399(34; 33, 36)	0(0; -)
Overweight	3441(37)	2833(82; 81, 84)	2707(79; 77, 80)	64(2; 1, 2)
Obese	1675(18)	1655(99; 98, 99)	1544(92; 91, 93)	861(51; 49, 54)
Education				
Illiterate	4538(49)	2471(55; 53, 56)	3083(68; 67, 70)	377(8; 8, 9)
Below diploma	4437(48)	2447(55; 54, 57)	2419(55; 53, 56)	506(11; 11, 12)
University	281(3)	168(60; 55, 66)	151(54; 48, 60)	42(15; 11, 20)
Marital status				
Not married	1049(11)	590 (57; 53, 59)	778 (75; 72, 77)	74 (7; 6, 9)
Married	8211(89)	4497(55; 54, 56)	4874 (60; 59, 61)	851 (10; 10, 11)
Socioeconomic status^a^				
Low	2419(26)	1095(46; 44, 48)	1405 (59; 57, 60)	165 (7; 6, 8)
Low-middle	2499(27)	1306(52; 51, 54)	1500 (60; 58, 62)	204 (8; 7, 9)
Middle-high	2046(22)	1166(57; 55, 59)	1270 (62; 60, 64)	234 (11; 10, 13)
High	2299(25)	1522(67; 65, 68)	1480 (65; 63, 67)	323 (14; 13, 16)
PA^b^				
Low	3061(33)	1906(63; 61, 64)	2143 (71; 69, 72)	367 (12; 11, 13)
Medium	3056(33)	1786(59; 57, 60)	1979 (65; 63, 67)	308 (10; 9, 11)
High	3146(34)	1397(45; 43, 46)	1533 (49; 47, 51)	251 (8; 7, 9)
Weight cycling				
Non cycler	7323(79)	4192(58; 56, 59)	4504(62; 61, 63)	867(12; 11, 13)
Cycler	1941(21)	897(46; 44, 49)	1151(60; 57, 62)	59(3; 2, 4)

BMI, Body mass index; CI, Confidence interval; PA, Physical activity; No, Number; WC, Waist circumference; WHR, Waist-hip ratio; WHtR, Waist-height ratio * Socioeconomic status (SES) was determined by assets analysis using multiple correspondence analysis of data on the properties. Several items were used to determine the SES of the participants, including phone at home, cell phone, washing machine, dishwasher, microwave device, camcorder, car, household residential area, number of rooms per household members, the main cooling devices and the main cooking device. Then participants were categorized into four ordered SES groups. † PA was assessed based on the Metabolic Equivalent of Task (MET) score. We implemented international physical activity questionnaire and used standard method for its analysis. Then, MET scores were divided into three thirties, named low, medium and high PA.

 WHtR had the highest correlation with pictogram scores followed by WC and WHR (*P* value < 0.001 in all pairs). Their corresponding pairs of Pearson’s correlation coefficients for males and females were (0.72, 0.78), (0.71, 0.73), and (0.56, 0.42), respectively. Table 2 shows the distribution of values for each anthropometric index across different pictogram scores.

**Table 2:** Gender Specific Median (IQR) of WC, WHR, and WHtR for Each Pictogram Score

**Male**					**BMI **(Blue, normal; Red, overweight; Green, obese)
**Score**	**No. (%)**	**WC (IQR)**	**WHR (IQR)**	**WHtR (IQR)**	
1	173 (4)	74 (70, 78)	0.86 (0.84, 0.90)	0.31 (0.29, 0.34)	**Figure:** 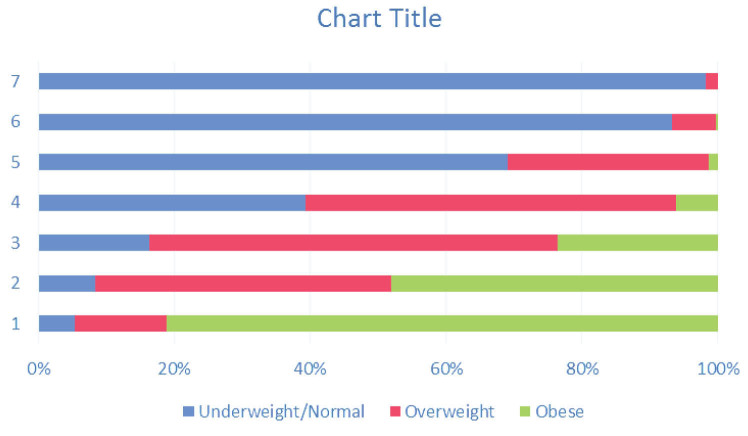
2	1081 (25)	79 (75, 86)	0.89 (0.85, 0.93)	0.35 (0.32, 0.38)	
3	1096 (26)	87 (81, 92)	0.93 (0.89, 0.97)	0.40 (0.37, 0.43)	
4	986 (23)	93 (87, 98)	0.96 (0.92, 0.99)	0.43 (0.40, 0.47)	
5	610 (14)	99 (94, 104)	0.99 (0.96, 1.02)	0.47 (0.44, 0.51)	
6	281 (7)	105 (99, 110)	1.02 (0.98, 1.05)	0.51 (0.47, 0.54)	
7	37 (1)	113 (108, 120)	1.04 (1.01, 1.07)	0.57 (0.51, 0.63)	
**Female**					**BMI **(Blue, normal; Red, overweight; Green, obese)
**Score**	**No. (%)**	**WC (IQR)**	**WHR (IQR)**	**WHtR (IQR)**	
1	239 (5)	74 (67, 80)	0.87 (0.81, 0.93)	0.30 (0.28, 0.33)	**Figure:** 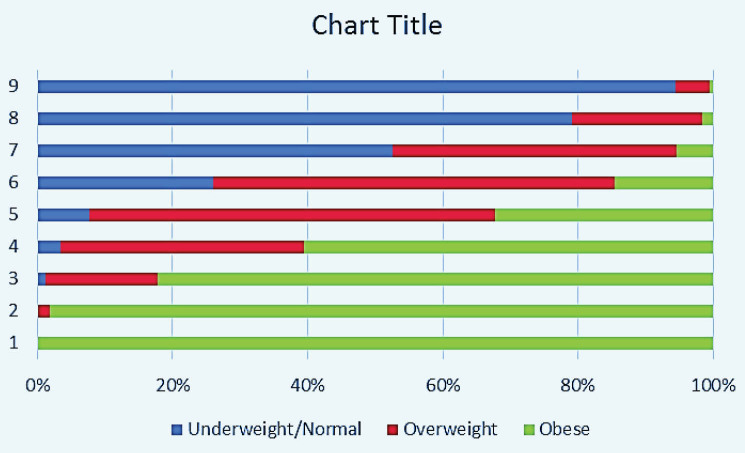
2	744 (15)	83 (76, 89)	0.91 (0.85, 0.98)	0.35 (0.32, 0.38)	
3	903 (18)	88 (81, 94)	0.94 (0.88, 0.99)	0.38 (0.35, 0.41)	
4	1066 (21)	93 (88, 100)	0.96 (0.91, 1.01)	0.41 (0.39, 0.44)	
5	964 (19)	97 (93, 103)	0.98 (0.93, 1.03)	0.45 (0.42, 0.48)	
6	599 (12)	103 (98, 107)	1.00 (0.95, 1.05)	0.48 (0.45, 0.51)	
7	322 (6)	108 (103, 113)	1.02 (0.97, 1.08)	0.52 (0.48, 0.55)	
8	115 (2)	113 (108, 121)	1.03 (0.97, 1.10)	0.56 (0.51, 0.60)	
9	25 (1)	123 (120, 132)	1.07 (1.04, 1.12)	0.64 (0.59, 0.66)	

IQR, Interquartile range; No, Number; WC, Waist circumference [measured in centimeters]; WHR, Waist-hip ratio; WHtR, Waist-height ratio.

**Figure 2:**
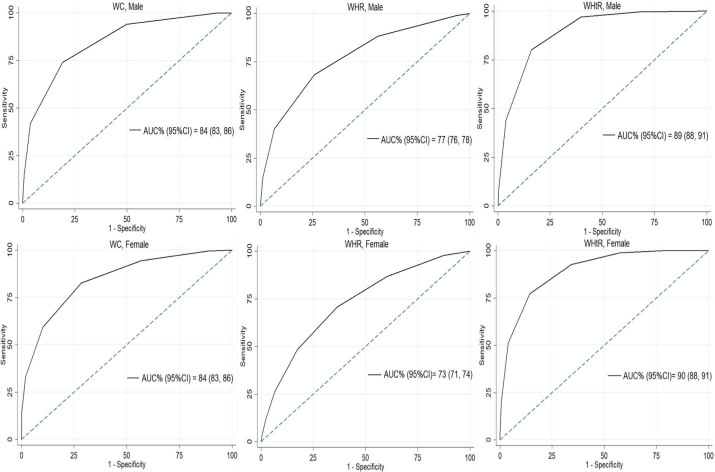
ROC Curves of Pictogram Scores to Classify At-Risk Population Based on Abdominal Obesity Using WC, WHR, and WHtR as Gold Standard Measures. Classification accuracy is represented as the area under the curve (AUC). ROC, receiver operating characteristic; WC, waist circumference; WHR, waist-hip ratio; WHtR, waist-height ratio.

 The estimated AUCs of pictogram scores to classify normal/at-risk abdominal obesity status for males and females were (84% [95% CI: 83, 86], 84% [95% CI: 83, 86]) for WC, (77% [95% CI: 76, 78], 73% [95% CI: 71, 74]) for WHR, and (89% [95% CI: 88, 91], 90% [95% CI: 88, 91]) for WHtR, respectively (Table 3 and Figure 2).

**Table 3:** Validity Measures of the Optimal Pictogram Cut-off Values to Classify At-Risk Population Based on WC, WHR, and WHtR

**Gender**	**Index**	**TP+FN (%)**	**FP+TN (%)**	**AUC% (95% CI)**	Cut-off*	**Sen% (95% CI)**	**Spe% (95% CI)**
Male							
	WC	1991 (46.90)	2254 (53.10)	84 (83, 86)	4	74 (72, 76)	81 (79, 82)
	WHR	1908 (44.95)	2337 (55.05)	77 (76, 78)	4	68 (66, 70)	74 (72, 76)
	WHtR	373 (8.79)	3872 (91.21)	89 (88, 91)	5	80 (76, 84)	84 (83, 85)
Female							
	WC	3092 (62.33)	1869 (37.67)	84 (83, 86)	4	83 (81, 84)	72 (69, 74)
	WHR	3741 (75.41)	1220 (24.59)	73 (71, 74)	4	71 (69, 72)	64 (61, 66)
	WHtR	552 (11.13)	4409 (88.87)	90 (88, 91)	6	77 (74, 81)	86 (84, 87)

AUC, Area under curve; FP, False positive; FN, False negative; TP, True positive; TN, True negative; Sen, Sensitivity; Spe, Specificity; CI, Confidence interval; WC, Waist circumference; WHR, Waist-hip ratio; WHtR, Waist-height ratio *Optimal pictogram cut-off value is the pictogram number with the highest AUC to classify at-risk from normal population

 Using WC, WHR, and WHtR as gold standard measures to define central obesity resulted in pictogram score cut-off values of 4, 4, and 5 in males and 4, 4, and 6 in females, respectively (Table 3 and Table S4). In males, the sensitivity and specificity values based on the optimal cut-off values were 74% and 81% for WC, 68% and 74% for WHR, and 80% and 84% for WHtR, respectively. The corresponding values in females were 83% and 72% for WC, 71% and 64% for WHR, and 77% and 86% for WHtR, respectively.

 Most of the individual characteristics did not have a statistically significant association with the accuracy of pictogram scores to classify central obesity. For both males and females, SES, PA, age group, ethnicity, marital status, and education had no effects on the classification accuracy of pictograms to determine central obesity. However, weight cycling had a significant association with the accuracy of pictogram scores classifying central obesity defined by WC and WHR (*P* value < 0.001), but not with WHtR (*P* value < 0.001 for both; Table 4).

**Table 4:** Effects of Sociodemographic Characteristics of the Study Population on the Accuracy of the Pictogram to Classify At-Risk Population Based on WC, WHR, and WHtR

	**WC**	**WHR**	**WHtR**
**Male**			
SES			
PA			
Cycling			
Age group			
Ethnicity			
Marital status			
Education			
**Female**			
SES			
PA			
Cycling			
Age group			
Ethnicity			
Marital status			
Education			

WC, waist circumference; WHR, waist-hip ratio; WHtR, waist-height ratio; SES, socioeconomic status; PA, Physical activity. Green, yellow, and red cells indicate *P *value ≥ 0.05, 0.001 ≤ *P *value < 0.05, and *P *value < 0.001, respectively.

 Sensitivity analysis was done based on the cut-off of abdominal obesity indices defined by the WHO and is presented in Supplementary file 1.

## Discussion

 The present study confirms the validity of using Stunkard’s set of pictograms to determine the population at risk of developing adverse health outcomes due to central obesity in the Iranian adult population. In both males and females, the highest AUC for predicting adverse health outcomes with pictograms was achieved when defining central obesity based on WHtR (0.89, 0.90), WC (0.84, 0.84), and WHR (0.77, 0.73), respectively. We also demonstrated the optimal cut-off for differentiating at-risk persons for each central obesity indices. Also, we have shown that the validity of pictograms is not affected by SES, PA, age group, ethnicity, marital status, and education; however, weight cycling influenced this validity when defining central obesity with WC and WHR in both genders.

 Most of the previous studies on the assessment of the accuracy of pictograms have focused on BMI. Pictograms were validated to classify obesity based on BMI with AUCs of 0.93 for females and 0.88 for males in the Caucasian population.^22^ Another study in the Japanese population validated the use of body shape silhouettes to discriminate between obese and thin individuals based on BMI with AUCs of > 0.80 in both genders.^23^ Lo et al^24^ found Stunkard’s pictograms to be superior to self-reported WC and waist to height ratio in the assessment of weight status (AUCs of > 0.8 in both genders) in Chinese adolescents. In the young healthy Spanish population, pictograms were validated to determine weight status defined by BMI with AUCs of 0.9 and 0.8 for males and females, respectively.^25^ Additionally, body silhouettes are shown to be reflective of past obesity based on measured and self-reported BMI in the European population.^10^ Body show cards were also validated to determine crude (based on BMI) and central (based on WC, WHtR) obesity in the African population with high accuracy (AUCs of > 0.9 in both genders).^26^ To date, only one Iranian study is conducted on the validity of pictograms to classify obesity. This study shows that Stunkard’s pictograms are valid to determine crude obesity (defined by BMI) in the Iranian population.^9^ Our study validates the accuracy of using the same set of pictograms to determine central obesity in the Iranian population with AUCs of 77% to 89% in males and 73% to 90% in females for different indices of central obesity.

 There is great variance in the age-standardized prevalence of obesity in our population using different definitions of central obesity (WC, WHR, or WHtR). Each index evaluates central obesity from a different perspective, and the optimal cut-off values for the classification of at-risk groups of the population using these indices are not necessarily obtained based on the same outcomes. Thus, an individual may be classified as at-risk according to a certain index while categorized as normal using another one at the same time. Despite being generally valid to classify central obesity, pictograms have higher accuracy to classify at-risk groups when central obesity is defined based on WHtR and WC rather than WHR. This finding results from higher pictogram score correlations with WHtR and WC than its correlation with WHR. This is the first study to assess the accuracy of pictograms to determine central obesity defined by WHR. A previous study in the African population revealed high accuracy (AUCs > 0.90) for the classification of central obesity defined by WHtR and WC using a different set of body show cards.^26^ These findings propose the currently developed body silhouettes as valid tools to evaluate central obesity defined by WHtR and WC. However, the results may not be readily translated to other measures of central adiposity like WHR. An underlying reason may be attributed to the inherent nature of the currently available show cards with more focus on horizontal and vertical dimensions of pictograms to demonstrate obesity rather than highlighting the ratio of waist to hip. Besides, most people may perceive obesity to be related to the degree of abdominal circumference or its ratio to height rather than other existing definitions of obesity like WHR.

 Previous studies report that sociodemographic and psychological factors are associated with self-perception of weight status. Dorsey et al^27^ found that weight misperception is highly prevalent among the US population and is associated with ethnicity and educational status. In another study, married women were shown to be more likely to perceive themselves as overweight while such an association was not found in men.^28^ Moreover, evidence suggests that the level of education affects the self-perception of obesity in the Korean adult population.^29^ In addition, another study including 5440 US adult population shows that race, SES, and level of education are associated with self-perception of being overweight.^30^ On the other hand, investigators demonstrated that weight misperception is associated with psychological distress and anxiety in the Australian and Chinese populations.^31,32^ Thus, there is the possibility that misperception of weight status affects the accuracy of self-reported body silhouettes to determine central obesity. Our results indicate that the utility of this instrument is not greatly influenced by the sociodemographic properties of individuals.

 SES, physical activity, age group, ethnicity, marital status, and level of education did not affect the validity of body silhouettes to classify central obesity. Although previous studies found an association between these factors and self-perception of weight, almost all of them have used self-reported height and weight or asked whether the participants have the feeling of being overweight.^27-30,33,34^ Selecting the closest silhouette to body shape appears to be more realistic and less prone to subjective errors observed with previous study designs. Most of our population belonged to Persian and Turk ethnicity, two major ethnic groups living in Iran. Gender-specific validity of body show cards to determine central obesity was almost the same between different ethnicities. In cases of large differences in performance, there might be a necessity to design new sets of body show cards that more closely resemble the morphologies observed in specific ethnicities. However, the results of our study are in favor of the generalizability of Stunkard’s pictograms to detect central obesity among different Iranian ethnicities. Alterations in body appearance may occur as a result of aging.^35^ Accordingly, we tested the potential influence of age group on the accuracy of pictograms classifying central obesity. Our results showed an almost similar classification accuracy among different age groups that support using the same set of pictograms across different age groups of the adult population. However, our study population was older than 40 years; the age group in which people’s (especially females’) perception of their weight status is probably less affected by social values and individual expectations.^36^ This may result in less bias and a more realistic interpretation of anthropometric status than that observed in a younger age group. Although weight cycling is used to point to previous fluctuations in weight status,^37-41^ it is a term with relative meaning depending on the context in which it has been described. Previous studies on weight cycling have mostly focused on its psychosocial and metabolic consequences.^38-40,42,43^ Overall, there has been controversy on the association of weight cycling with the risk of adverse metabolic outcomes^39,43^; however, an association has been shown between a history of weight cycling and central fat accumulation in the body.^38,44^ Furthermore, previous evidence suggests the association between the history of weight cycling and the accuracy of self-reported weight status.^41^

 Our findings indicate that the history of weight cycling negatively impacts the performance of body pictograms to accurately predict central obesity status. Hence, caution should be made on the interpretation of pictogram ratings in those who report previous fluctuations in their weight. It can be assumed that those with weight cycling were probably not satisfied with their actual weight. The discrepancy between one’s actual and ideal body weight as well as the effects of weight cycling on central adiposity can both account for the decreased accuracy of pictograms in cyclers.

 To date, this is one of the largest studies using the pictogram ratings for the evaluation of obesity in the Iranian population. The population-based nature of our study with more than 9000 participants and adherence to precise measurement protocols make our results generalizable to other nations in developing countries. However, this study has certain limitations. First, our study population was older than 40 years and future studies are needed to assess the validity of body show cards in children and young adults. Second, this study was conducted in a semi-urban area; thus, the sociodemographic characteristics of our population may not be fully representative of those living in high social classes of urban areas. Moreover, participants self-determined the closest body show card to their actual body shape. There has been a higher correlation between pictogram scores selected by expert anthropometry observers than those selected by one expert and one less skilled observer.^45^ However, our results have better generalizability to the situations where individuals have to self-determine their scores with implications in large studies and telemedicine.

 In conclusion, the pictogram is a valid tool to classify central obesity in the adult population and the ratings can be better interpreted with respect to the sociodemographic context of the target population. This sets a framework to access anthropometric information on a large scale that would have been otherwise difficult to obtain due to the problems associated with human resources, device expenses, and lack of direct access to people.

## Supplementary Materials

Supplementary file 1 contains Tables S1-S4.
