# Supplementary material for: Pictogram Is a Valid Instrument to Classify At-Risk Adult Population Based on Abdominal Obesity: Results from Pars Cohort Study
Source: Arch Iran Med. 2022 Jun 1;25(6):366–74. doi: 10.34172/aim.2022.60 (PMC11904271; doi:10.34172/aim.2022.60)
Supplement: Supplementary file 1 — contains Tables S1-S4. [file aim-25-366-s001.pdf]

Supplementary file 1

**Table S1.** Demographics of the overall and at-risk population based on WC, WHR, and WHtR				
**Variables**	**Overall (*n* = 9264) No (%)**	**At Risk**		
		**WC No (%; 95%CI)**	**WHR No (%; 95%CI)**	**WHtR No (%; 95%CI)**
**Age**				
Mean age (SD)	52.65(9.70)	52.65(9.33)	53.08(9.65)	50.86(8.12)
<50	4216(46)	1705(40; 39, 42)	3193(76; 75,77)	205(5; 4, 6)
50–59	2808(30)	1175(42; 40, 44)	2295(82; 81, 84)	141(5; 4, 6)
>59	2240(24)	944(42; 40, 44)	1887(85; 83, 86)	66(3; 2, 4)
**Gender**				
Female	4987(54)	3300(66; 65, 67)	4413(89; 88, 90)	270(5; 5, 6)
Male	4276(46)	524(12; 11, 13)	2962(70; 68, 71)	141(3; 3, 4)
**Ethnicity**				
Persian	5216(56)	2274(44; 42, 45)	4234(82; 80, 83)	273(5; 5, 6)
Turk	3596(39)	1341(37; 36, 39)	2758(77; 76, 79)	119(3; 3, 4)
Other	451(5)	209(46; 42, 51)	383(85; 82, 88)	19(4; 3, 7)
**BMI**				
Underweight/Normal	4091(44)	320(8; 7, 9)	2442(60; 58, 61)	0(0; -)
Overweight	3441(37)	1871(54; 53, 56)	3280(95; 95, 96)	0(0; -)
Obese	1675(18)	1583(95; 93, 96)	1644(98; 97, 99)	364(22; 20, 24)
**Education**				
Illiterate	4538(49)	2209(49; 47, 50)	3754(83; 82, 84)	184(4; 4, 5)
Below diploma	4437(48)	1556(35; 34, 36)	3384(77; 75, 78)	206(5; 4, 5)
University	281(3)	57(20; 16, 25)	231(83; 78, 87)	19(7; 4, 10)
**Marital status**				
Not married	1049(11)	598(57; 54, 60)	899(86; 84, 88)	35(3; 2, 5)
Married	8211(89)	3224(39; 38, 40)	6473(79; 78, 80)	376(5; 4, 5)
**Socioeconomic status***				
Low	2419(26)	906(37; 36, 39)	1834(76; 75, 78)	80(3; 3, 4)
Low-Middle	2499(27)	996(40; 38, 42)	1957(79; 77, 80)	91(4; 3, 4)
Middle-High	2046(22)	862(42; 40, 44)	1655(81; 79, 83)	90(4; 4, 5)
High	2299(25)	1060(46; 44, 48)	1929(84; 83, 86)	150(7; 6, 8)
**PA**				
Low	3061(33)	1593(52; 50, 54)	2633(87; 85, 88)	176(6; 5, 7)
Medium	3056(33)	1414(46; 45, 48)	2555(84; 83, 85)	135(4; 4, 5)
High	3146(34)	817(26; 24, 28)	2187(70; 68, 71)	100(3; 3, 4)
**Weight Cycling**				
Non cycler	7323(79)	3079(42; 41, 43)	5849(80; 79, 81)	388(5; 5, 6)
Cycler	1941(21)	745(38; 36, 41)	1526(79; 77, 81)	24(1; 1, 2)

BMI, body mass index; CI, confidence interval; PA, physical activity; No, number; WC, waist circumference; WHR, waist-hip ratio; WHtR, waist-height ratio
*Socioeconomic status improves from 1 to 4
WHO-proposed cut-offs of WC > 102 cm, WHR > 0.9 in men; WC > 88 cm, WHR > 0.85 in women and WHtR ≥ 0.55 were used to define at-risk subgroup of individuals with abdominal obesity.

Table S2. Validity measures of the optimal pictogram cut-off values to classify at-risk population based on WC, WHR, and WHtR

**Gender**	**Index**	**TP+FN (%)**	**FP+TN (%)**	**AUC%(95% CI)**	**Cut-off***	**Sen%(95%CI)**	**Spe%(95%CI)**
Male							
	WC	524 (12.25)	3752 (87.75)	88 (86, 89)	5	76 (72, 80)	86(84, 87)
	WHR	2962 (69.60)	1294 (30.40)	78 (77, 79)	3	83 (82, 85)	58(56 ,61)
	WHtR	141 (3.30)	4135 (96.70)	86 (82, 89)	5	79(72, 86)	80(79, 81)

Female							
	WC	3300 (66.17)	1687 (33.83)	84 (83, 85)	4	81(79, 82)	74(72, 76)
	WHR	4413 (88.95)	548 (11.05)	77 (75, 79)	4	67 (66, 68)	77(72, 80)
	WHtR	270 (5.41)	4717 (94.59)	88 (85, 90)	6	83(78, 88)	82(81, 83)

AUC, area under curve; FP, false positive; FN, false negative; TP, true positive; TN, true negative; Sen, sensitivity; Spe, specificity; CI, confidence interval; WC, waist circumference; WHR, waist-hip ratio; WHtR, waist-height ratio
*Optimal pictogram cut-off value is the pictogram number with the highest AUC to classify at-risk from normal population
WHO-proposed cut-offs of WC > 102 cm, WHR > 0.9 in men; WC > 88 cm, WHR > 0.85 in women and WHtR ≥ 0.55 were used to define at-risk subgroup of individuals with abdominal obesity

.

Table S3. Effects of sociodemographic characteristics of the study population on the accuracy of the pictogram to classify at-risk population based on WC, WHR, and WHtR

	WC	WHR	WHtR
		**Male**	
**SES**			
**PA**			
**Cycling**			
**Age group**			
**Ethnicity**			
**Marital status**			†
**Education**			
		**Female**	
**SES**			
**PA**			
**Cycling**			
**Age group**			
**Ethnicity**			
**Marital status**			
**Education**			† †

WC, waist circumference; WHR, waist-hip ratio; WHtR, waist-height ratio; SES, socioeconomic status; PA, Physical activity.
Green, yellow, and red cells indicate *P*-value >= 0.05, 0.001<= *P*-value <0.05, and *P*-value < 0.001, respectively
†Omitted due to low number of at-risk unmarried participants
††Those with university degree were omitted from the analysis due to low number of participants in this category
WHO-proposed cut-offs of WC > 102 cm, WHR > 0.9 in men; WC > 88 cm, WHR > 0.85 in women and WHtR ≥ 0.55 were used to define at-risk subgroup of

individuals with abdominal obesity.

**Table S4.** Sensitivity and specificity of different pictogram cut-off values to classify normal/at-risk population based on WC, WHR, and WHtR				
**Gender**	**Index**	**Cut-off**	**Sensitivity%**	**Specificity%**
**Male**				
	WC			
		2	100%	7%
		3	94%	50%
		4	74%	81%
		5	42%	96%
		6	15%	99%
	WHR			
		2	99%	7%
		3	88%	44%
		4	68%	74%
		5	40%	93%
		6	15%	99%
	WHtR			
		2	100%	4%
		3	100%	32%
		4	97%	60%
		5	80%	84%
		6	44%	96%
**Female**				
	WC			
		2	99%	12%
		3	94%	43%
		4	83%	72%
		5	59%	90%
		6	33%	98%
		7	15%	100%
		8	4%	100%
	WHR			
		2	98%	12%
		3	87%	40%
		4	71%	64%
		5	48%	83%
		6	26%	94%
		7	12%	98%
		8	4%	100%
	WHtR			
		2	100%	5%
		3	100%	22%
		4	99%	42%
		5	93%	66%
		6	77%	86%
		7	51%	96%
		8	20%	99%
